# Supplementary material for: Viral miRNA adaptor differentially recruits miRNAs to target mRNAs through alternative base-pairing
Source: eLife. 2019 Sep 20;8:e50530. doi: 10.7554/eLife.50530 (PMC6763288; doi:10.7554/eLife.50530)
Supplement: Supplementary file 5. [file elife-50530-supp5.docx]

| **Key Resources Table** | | | | |
| --- | --- | --- | --- | --- |
| **Reagent type (species) or resource** | **Designation** | **Source or reference** | **Identifiers** | **Additional information** |
| cell line  (*Callithrix jacchus*) | T lymphocytes transformed by *Herpesvirus saimiri* strain A11  cj319-WT | Cook et al., 2004 |  |  |
| cell line  (*Callithrix jacchus*) | T lymphocytes transformed by *Herpesvirus saimiri* strain A11  cj137-WT | Gorbea et al., 2017 |  |  |
| cell line  (*Homo sapiens*) | 293T/17  Epithelial embryonic kidney | ATCC | Cat# CRL-11268, RRID:CVCL_1926 |  |
| cell line  (*Homo sapiens*) | U937  Adult acute monocytic leukemia | ATCC | Cat# CRL-1593.2, RRID:CVCL_0007 |  |
| recombinant DNA reagent | pGL4.54 (plasmid) | Promega | Cat# E5061  GenBank Accession Number KM359769 | Used to obtain the thymidine kinase promoter cloned into pLenti-CMVTRE3G-eGFP-Puro |
| recombinant DNA reagent | pmirGLO (plasmid) | Promega | Cat# E1330  GenBank Accession Number FJ376737 | Used to obtain the optimized *luc2* and *Renilla* luciferase genes cloned into pLenti-CMVTRE3G-eGFP-Puro and LeGO-G/BSD, respectively |
| recombinant DNA reagent | pcDNA3.1  (plasmid) | ThermoFisher | Cat# V79520 | Used to obtain the bovine growth hormone (bGH) polyadenylation signal cloned into pLenti-CMVTRE3G-eGFP-Puro |
| recombinant DNA reagent | pLenti-CMVTRE3G-eGFP-Puro (w819-1) | Addgene | RRID:Addgene_27570 | Vector backbone used to generate pLenti-TK-Firefly-Control plasmid |
| recombinant DNA reagent | pLenti-TK-Firefly-Control (plasmid) | Addgene | RRID:Addgene_122277 | Vector backbone used to generate luciferase reporters used in this study |
| recombinant DNA reagent | pLenti-TK-Firefly-cjBCCIP  (plasmid) | Addgene | RRID:Addgene_122278 | Full-length *Callithrix jacchus* BCCIP 3'UTR cloned between the *luc2* gene and bGH polyA signal of pLenti-TK-Firefly-Control |
| recombinant DNA reagent | pLenti-TK-Firefly-cjCD69  (plasmid) | Addgene | RRID:Addgene_122279 | Partial *Callithrix jacchus* CD69 3'UTR cloned between the *luc2* gene and bGH polyA signal of pLenti-TK-Firefly-Control |
| recombinant DNA reagent | pLenti-TK-Firefly-cjMGA  (plasmid) | Addgene | RRID:Addgene_122280 | Partial *Callithrix jacchus* MGA 3'UTR cloned between the *luc2* gene and bGH polyA signal of pLenti-TK-Firefly-Control |
| recombinant DNA reagent | pLenti-TK-Firefly-cjPACS1  (plasmid) | Addgene | RRID:Addgene_122281 | Partial *Callithrix jacchus* PACS1 3'UTR cloned between the *luc2* gene and bGH polyA signal of pLenti-TK-Firefly-Control |
| recombinant DNA reagent | pLenti-TK-Firefly-cjSTK4  (plasmid) | Addgene | RRID:Addgene_122282 | Partial *Callithrix jacchus* STK4 3'UTR cloned between the *luc2* gene and bGH polyA signal of pLenti-TK-Firefly-Control |
| recombinant DNA reagent | pLenti-TK-Firefly-cjTP53RK  (plasmid) | Addgene | RRID:Addgene_122283 | Partial *Callithrix jacchus* TP53RK 3'UTR cloned between the *luc2* gene and bGH polyA signal of pLenti-TK-Firefly-Control |
| recombinant DNA reagent | pLenti-TK-Firefly-cjYTHDC1  (plasmid) | Addgene | RRID:Addgene_122284 | Partial *Callithrix jacchus* YTHDC1 3'UTR cloned between the *luc2* gene and bGH polyA signal of pLenti-TK-Firefly-Control |
| recombinant DNA reagent | LeGO-G/BSD  (plasmid) | Addgene | RRID:Addgene_27354 | Cloning vector for *Renilla* luciferase gene |
| recombinant DNA reagent | LeGo-Renilla-BLAST  (plasmid) | Addgene | RRID:Addgene_122276 | Lentiviral targeting vector for expression of *Renilla* luciferase |
| recombinant  DNA  reagent | pBS-GFP-HSUR2  (plasmid) | Gorbea et al., 2017  PMID: 28976967 |  |  |
| recombinant DNA reagent | pBS-GFP-ΔHSUR2  (plasmid) | Gorbea et al., 2017  PMID: 28976967 |  |  |
| recombinant DNA reagent | pBS-GFP-HSUR2Δ142-3p  (plasmid) | Gorbea et al., 2017  PMID: 28976967 |  |  |
| recombinant DNA reagent | pMD2.G  (plasmid) | Addgene | RRID:Addgene_12259 | Lentivirus packaging vector |
| recombinant DNA reagent | pMDLG/pRRE  (plasmid) | Addgene | RRID:Addgene_12251 | Lentivirus packaging vector |
| recombinant DNA reagent | pRSV-Rev  (plasmid) | Addgene | RRID:Addgene_12253 | Lentivirus packaging vector |
| commercial assay or kit | oligo(dT)25 magnetic beads | New England BioLabs | S1419 |  |
| commercial assay or kit | Dynabeads™ MyOne™ Streptavidin C1 | ThermoFisher | Cat# 65002 |  |
| commercial assay or kit | SMARTer Stranded Total RNA-seq v2—Pico Input Mammalian | Takara Bio | Cat# 635006 |  |
| commercial assay or kit | Amaxa human T-cell | Lonza | cat# VPA-1002 |  |
| commercial assay or kit | Amaxa kit V | Lonza | cat# VCA-1003 |  |
| commercial assay or kit | High Capacity cDNA Reverse Transcription | Applied Biosystems | cat# 4368814 |  |
| commercial assay or kit | Dual-luciferase reporter assay system | Promega | cat# E1980 |  |
| commercial assay or kit | SYBR-fast qPCR Master Mix optimized for Roche LightCycler 480 | Roche | KK4611 |  |
| chemical compound, drug | 4’-aminomethyl-4,5’,8-trimethylpsoralen | Cayman Chemical | Cat# 17162 |  |
| chemical compound, drug  (enzyme) | S1 nuclease | ThermoFisher | Cat# EN0321 |  |
| chemical compound, drug  (enzyme) | RNase-H | New England BioLabs | Cat# M0297S |  |
| chemical compound, drug  (enzyme) | RNase-free DNase I | New England BioLabs | Cat# M0303L |  |
| chemical compound, drug | mirVana™ miRNA inhibitor negative control #1 | Ambion | cat# 4464076 |  |
| chemical compound, drug | hsa-miR-142-3p miRCURY LNA inhibitor | Exiqon | cat# 4100271-001 |  |
| chemical compound, drug | hsa-miR-16-mirVana | Ambion | cat# 4464084 |  |
| software, algorithm | STAR (v2.5.3a) |  | RRID:SCR_015899  Dobin et al. 2013  https://doi.org/10.1093/bioinformatics/bts635 |  |
| software, algorithm | DESeq2 (v1.14.1) |  | RRID:SCR_015687  Love et al. 2014  doi:10.1186/s13059-014-0550-8 |  |
| software, algorithm | RNAcofold | <http://rna.tbi.univie.ac.at/cgi-bin/RNAfold.cgi> | RRID:SCR_008550  Lorenz et al. 2011  PMID: 22115189 |  |
| software, algorithm | RNAfold | <http://rna.tbi.univie.ac.at/cgi-bin/RNAfold.cgi> | RRID:SCR_008550  Lorenz et al. 2011  PMID: 22115189 |  |
| software, algorithm | GOrilla | <http://cbl-gorilla.cs.technion.ac.il/> | RRID:SCR_006848  Eden et al 2009  <https://doi.org/10.1186/1471-2105-10-48> |  |
| software, algorithm | MEME analysis suite | <http://meme-suite.org/> | RRID:SCR_001783  Bailey et al. 2009  <https://doi.org/10.1093/nar/gkp335> |  |
| software, algorithm | RSAT | <http://rsat.ulb.ac.be/rsat/> | RRID:SCR_008560  van Helden et al. 1998  PMID: 9719638 |  |
